# Characteristics of the Inflammatory Bowel Disease in Children: A Croatian Single-Centre Retrospective Study

**DOI:** 10.3390/children10101677

**Published:** 2023-10-11

**Authors:** Ivan Pivac, Antonia Jelicic Kadic, Ranka Despot, Vanda Zitko, Darija Tudor, Edita Runjic, Josko Markic

**Affiliations:** 1 School of Medicine, University of Split, Soltanska 2, 21000 Split, Croatia; ivan.pivac4@gmail.com; 2 Department of Pediatrics, University Hospital of Split, Spinciceva 1, 21000 Split, Croatia; ajelkad@kbsplit.hr (A.J.K.); rdespot@kbsplit.hr (R.D.); dtudor@kbsplit.hr (D.T.); erunjic@kbsplit.hr (E.R.)

**Keywords:** inflammatory bowel disease, IBD, ulcerative colitis, Crohn’s disease, child, pediatric

## Abstract

Inflammatory bowel diseases (IBDs), encompassing ulcerative colitis (UC) and Crohn’s disease (CD), are chronic gastrointestinal disorders often diagnosed in youth, presenting unique features compared to adult-onset cases. We aimed to profile pediatric IBD patients in Croatia through a retrospective analysis of children up to 18 years old diagnosed with IBD at the University Hospital of Split from 1 January 2012, to 31 December 2021, utilizing data collected during hospitalization for diagnosis. Over a decade, 107 children were diagnosed, with 43.9% having UC, 55.1% CD, and 0.9% IBD-unclassified. Median age at diagnosis was 14.1 years, with UC patients being older (14.8 vs. 13.7 years, *p* = 0.044). Males constituted 60.7% of patients. Median symptom duration was 2.0 months, with CD patients experiencing a longer diagnostic delay (3.0 vs. 2.0 months, *p* = 0.003). The median incidence rate was 9.89 (95% CI 5.93–13.84) per 100,000 children/year, varying across age groups. Median (IQR) BMI z-score was −0.34 (−0.97–0.45). Common symptoms included diarrhea (60.7%) and abdominal pain (50.5%), with rectal bleeding more prevalent in UC (72.3% vs. 32.2%, *p* < 0.001). While our study offers valuable insights into pediatric IBD in Croatia, further prospective research is needed to clarify disease progression and development.

## 1. Introduction

Inflammatory bowel disease (IBD) comprises chronic inflammatory conditions affecting the digestive system, including Crohn’s disease (CD) and ulcerative colitis (UC) [1]. These conditions are distinguished through clinical, endoscopic, histological, and radiological criteria, with fewer than 10% of cases categorized as indeterminate colitis [2]. UC primarily causes inflammation and ulcers in the colon and rectum [3], while CD exhibits a segmental distribution, affecting the entire gastrointestinal tract from mouth to anus and potentially leading to transmural inflammation and fistula [3,4].

In recent decades, IBD has become a growing public health concern [5]. The global incidence and prevalence of CD and UC vary significantly, influenced by factors like geographic location, environmental conditions, immigration patterns, and ethnic disparities. Although UC was previously believed to be more prevalent, recent years have seen CD become increasingly predominant [6]. In North America and Europe, over 3.5 million people are affected by IBD. The highest prevalence is observed in Norway, Germany, the United States, and Canada, where IBD affects over 0.3% of the population. Data on IBD prevalence in Croatia also vary, ranging from less than 0.1% to approximately 0.25% [5].

The precise cause of IBD remains elusive, but it is thought to result from an abnormal immune response to gut microbes, influenced by genetic and environmental factors. While genetic studies have identified susceptibility gene loci, they only partially explain the disease’s variability, indicating complex interactions between genetics, microbes, and the environment in IBD development. Recent research has emphasized the role of both innate and adaptive immune responses in gut inflammation [4].

Symptoms of IBD exhibit complexity and considerable variation [4,7]. Common clinical symptoms such as abdominal pain and diarrhea, which are equally prevalent in both UC and CD, can sometimes resemble those observed in conditions like irritable bowel syndrome, allergic gastroenteritis, or infectious gastroenteritis. Furthermore, rectal bleeding is more frequent in UC, occurring in over 90% of cases, whereas only 40% of CD patients experience this symptom. Conversely, individuals with CD often manifest weight loss and perianal disease [7]. Individuals with IBD may experience additional non-gastrointestinal (GI) symptoms, including fatigue, depression, and anxiety. Both GI and non-GI symptoms can lead to disruptions in daily life, such as reduced participation in social activities, compromised work productivity, and a diminished overall quality of life [8].

In addition to GI symptoms and complications associated with IBD, some patients develop extra-intestinal manifestations. These manifestations may or may not respond to treatment of the underlying disease. Among the most common extra-intestinal manifestations are those involving the musculoskeletal system, skin, hepatobiliary tract, and eyes [7,9]. Individuals diagnosed with celiac disease (CeD), another significant chronic GI disorder, may also have a higher likelihood of developing IBD compared to the general population, and vice versa [10]. Consequently, effectively managing both primary symptoms and extra-intestinal manifestations in individuals with IBD is crucial for optimizing their overall health and well-being [8,9].

The diagnosis of IBD requires a comprehensive approach, beginning with a meticulous physical examination and a thorough evaluation of the patient’s personal and family medical history. However, the pivotal diagnostic tool in IBD is GI tract endoscopy [8,11]. For suspected cases in children, the initial diagnostic steps involve ileocolonoscopy and esophagogastroduodenoscopy (EGD). During these procedures, it is essential to collect two or more biopsies from each visualized section of the GI tract, even when no macroscopic lesions are evident [3,11,12,13]. Additionally, fecal calprotectin, a biomarker renowned for its high sensitivity and specificity, has gained increasing significance. It serves as a discriminating factor between inflammatory and non-inflammatory GI conditions and can provide valuable insights into the disease progression in individuals with IBD [12,14].

The management of IBD is a complex, long-term, and costly endeavor, involving various therapeutic approaches [5,15,16]. Traditional treatments primarily focus on symptom control through pharmacotherapy, including aminosalicylates, corticosteroids, and immunomodulators, along with general measures, diet, and, when necessary, surgical resection. Recent advancements in IBD management have shifted treatment goals from solely improving daily symptom management to actively pursuing mucosal healing [15]. Patient education also plays a crucial role in enhancing the effectiveness of IBD treatment [15].

IBD exhibits two primary age-related incidence peaks: one occurring in younger adults between 20 and 40 years of age, and another between 60 and 70 years of age [6]. However, up to 30% of patients receive an IBD diagnosis during childhood or adolescence [17]. Recently, there has been an increase in the incidence of IBD among pediatric populations, primarily attributed to CD, with a higher prevalence among males than females [18]. Pediatric IBD cases display several distinct characteristics, differing significantly from adult-onset IBD. Furthermore, even within adult patients, those whose disease began in childhood differ from those diagnosed as adults [18].

Childhood IBD often presents with more extensive disease and a more aggressive course than adult-onset disease [17,18]. Within the pediatric IBD population, there is a one-third risk of needing surgery within first five years of disease. In addition to classic symptoms common in adults, pediatric cases often include more systemic symptoms like weight loss, anorexia, growth delay, delayed puberty, skin changes, depression, and anxiety [17].

In pediatrics, disease classification follows the revised Porto criteria, placing patients into one of five categories: typical UC, atypical UC, clear CD (including colonic CD), normal, or IBD–unclassified [13]. Particular attention should be directed toward aspects like the impact on growth and development, school absenteeism, quality of life, missed school days, reduced participation in extracurricular activities, subjective school underperformance, and therapy adherence. Surprisingly, research indicates that up to 50% of pediatric patients do not adhere adequately to therapy. The transition to adult care involves more than merely transferring medical records; it plays a pivotal role in developing lifelong care patterns [17].

Despite the growing body of research focused on IBD, particularly in the pediatric population, findings to date have failed to explain the differences in the characteristics of afflicted individuals across diverse geographic regions [17,18,19,20]. While some studies may exist regarding the characteristics of pediatric IBD patients in Croatia, to the best of the authors’ knowledge, none have provided a thorough and comprehensive analysis. Therefore, our research aims to offer an extensive overview of the attributes of children affected by this chronic, lifelong condition in Croatia. Our objective is to closely scrutinize the characteristics of these patients at the disease’s onset, ultimately advancing our understanding of its etiopathogenesis and potentially improving disease outcomes.

## 2. Materials and Methods

### 2.1. Study Design

This was a retrospective study conducted within the Department of Pediatrics at the University Hospital of Split, located in Split, Croatia.

### 2.2. Ethics

The research was carried out in accordance with all applicable guidelines aimed at ensuring proper conduct and participant safety, while adhering to the principles of the Helsinki Declaration [21]. All data were collected anonymously, without gathering personal or identifiable information. The study protocol received approval from the Ethics Committee of the University Hospital of Split, Croatia (protocol code: 2181-147/01/06/M.S.-22-02).

### 2.3. Participants

Children (aged < 18 years) who were diagnosed with IBD from 1 January 2012, to 31 December 2021, at the University Hospital of Split, Croatia, were eligible for inclusion in the analysis. The diagnoses of either CD, UC, or IBD unclassified (IBD-U) were established in accordance with the criteria outlined by the European Society for Pediatric Gastroenterology Hepatology and Nutrition [13]. This diagnostic process involved a comprehensive assessment, including an evaluation of family history, physical and laboratory examinations, endoscopy with histopathological assessment, and small bowel imaging [18].

### 2.4. Data Collection and Description

Medical records from the initial hospitalization during which IBD was diagnosed were reviewed. Comprehensive data encompassing demographic information, family histories of autoimmune disorders, clinical presentations, physical examinations, laboratory findings, diagnostic procedures, and therapeutic regimens were gathered and analyzed. The body mass index (BMI) was determined using the formula: weight/height^2^ (kg/m^2^), and z-scores for weight, height, and BMI were computed employing the Centers for Disease Control and Prevention (CDC) growth charts [22]. To categorize patients as severely underweight, underweight, normal weight, or overweight, specific BMI SD cut-off values were applied: ≤−3, ≤−2, −2–1, and >1, respectively [23,24]. For severe short stature, a height SD cut-off value of ≤−3 was employed [25]. Regarding comparison by age, patients were divided into groups: those younger than 6 years, termed very early onset IBD (VEO-IBD), and older patients, who were divided into two equal groups: 6–<12 years and 12 years or more [26].

Incidence rates per 100,000 children a year were calculated using population estimates from the Croatian Bureau of Statistics [27]. These incidence rates were specifically computed for Split-Dalmatia County, Croatia. The differentiation between urban and rural residency was based on the territorial division of the Republic of Croatia, where smaller administrative units (municipalities) were classified as rural, while larger ones (cities) were categorized as urban [28].

Age and sex-specific reference intervals for laboratory parameters were drawn from the Canadian Laboratory Initiative on Pediatric Reference Intervals (CALIPER) [29]. Assessment of vitamin D status adhered to the guidelines of the Central European Scientific Committee and the United States Endocrine Society, utilizing reference intervals for vitamin D (25O HD) as follows: deficiency (0–50 nmol/L), insufficiency (50–75 nmol/L), and target concentration (75–125 nmol/L) [30].

### 2.5. Statistical Analysis

The data were encoded and entered into Microsoft Excel spreadsheet version 16.0 (Microsoft Corporation, Redmond, WA, USA). Statistical analyses were performed using IBM SPSS Statistics version 23 (IBM Corp., Armonk, NY, USA). To calculate 95% confidence intervals (95% CI) for medians, we utilized MedCalc software version 22.009 (MedCalc Software, Ostend, Belgium). Categorical variables are presented as absolute numbers and proportions. Continuous variables, due to their non-normal distribution, are reported as medians with interquartile ranges (IQRs). Incidence rates were assessed using the median and a 95% CI. We employed the Chi-squared test for comparing categorical variables, the Mann–Whitney U test for comparing continuous variables, and the Kruskal–Wallis H test for comparisons involving more than two independent samples. A *p*-value < 0.05 was considered statistically significant.

## 3. Results

**Figure 1:**
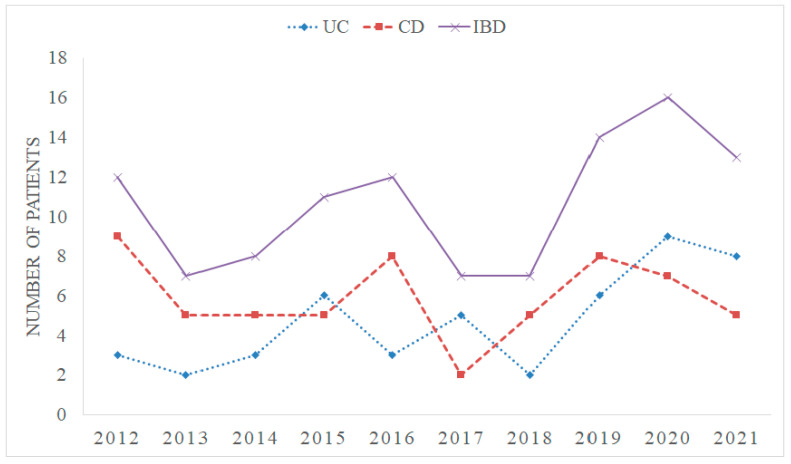
The time trends of total inflammatory bowel disease (IBD) diagnosed cases per year at University Hospital of Split, Croatia. UC: ulcerative colitis, CD: Crohn’s disease.

Between January 2012 and December 2021, a total of 107 children were diagnosed with IBD at our hospital. Among them, 47 (43.9%) patients were diagnosed with UC, 59 (55.1%) with CD, and 1 (0.9%) case was categorized as IBD-unclassified. The annual trends in the total number of diagnosed IBD cases are illustrated in Figure 1.

As indicated in Table 1, the median (IQR) age at the time of diagnosis was 14.1 (11.6–16.1) years. Patients diagnosed with UC were older compared to those with CD (14.8 (12.8–16.7) vs. 13.7 (11.4–15.8) years, *p* = 0.044). Two patients (1.9%) were under the age of 6 years, and both were diagnosed with very early-onset inflammatory bowel disease, with one patient being less than 2 years old at the time of diagnosis (infantile onset IBD). Both very early-onset IBD patients had UC.

When assessing defined age groups, a significantly higher proportion of patients diagnosed with UC were older than 12 years (80.9%), whereas approximately two-thirds of CD patients (67.8%) fell within the same age group, with one-third in the 6–<12 years age category (32.2%), *p* = 0.043.

Overall, 60.7% of the patients were males. Data regarding the duration of symptoms, available for 97 patients, showed a median (IQR) symptom duration of 2.0 (1.0–5.0) months. Patients diagnosed with CD experienced a significantly longer period from the onset of initial symptoms to diagnosis compared to UC patients (3.0 (2.0–6.0) vs. 2.0 (0.0–3.0) months, *p* = 0.003).

Nine patients (8.4%) had a family history of IBD among their first- and/or second-degree relatives, while nineteen patients (17.8%) had a positive family history of other autoimmune disorders, including diabetes mellitus, psoriasis, hypothyroidism, hyperthyroidism, Hashimoto’s thyroiditis, asthma, celiac disease, vitiligo, Addison’s disease, and juvenile idiopathic arthritis.

Among all IBD patients, 80 (74.8%) resided in Split-Dalmatia County, Croatia, for which specific incidence rates were calculated and are presented in Table 2. The median (95% CI) incidence rate per 100,000 children/year in Split-Dalmatia County was 9.89 (95% CI 5.93–13.84). A statistically significant difference in the incidence of IBD was observed when comparing age groups. The lowest incidence was recorded among those under 6 years of age, at 0.00 (95% CI 0.00–1.96), followed by those aged 6–<12 (5.75 (95% CI 3.52–10.73)), while the highest incidence was observed among the eldest group, at 19.68 (95% CI 11.87–26.42), *p* < 0.001.

Anthropometric measures which were available for 105 patients are presented in Table 3. There were no statistically significant differences in height, weight, or BMI z-scores between CD and UC patients.

The clinical presentation of patients is detailed in Table 4. In UC patients, the most prevalent symptoms included rectal bleeding, diarrhea, and abdominal pain, while CD patients predominantly presented with abdominal pain, diarrhea, and rectal bleeding, respectively. Additionally, the frequency of rectal bleeding was significantly higher in individuals with UC, *p* < 0.001.

Two patients (1.9%) who underwent appendectomy due to acute appendicitis were subsequently diagnosed with CD based on histopathological evaluation. One patient (0.9%) had Crohn’s disease complicated by an intra-abdominal abscess. Extra-intestinal manifestations were observed in 17.8% of patients. Interestingly, children residing in rural areas were more likely to have extra-intestinal manifestations compared to those in urban areas (33.3% vs. 13.0%, *p* = 0.015).

Approximately one-fourth of the patients (26.1%) had associated diseases, while allergies were reported in 12 (11.2%) patients. Perianal manifestations were present in 13.6% of CD patients, while none of the UC patients had such manifestations, *p* = 0.009.

The percentages of patients with abnormal laboratory parameters, based on available measurements, are summarized in Table 5. More than two-thirds of patients (71.3%) exhibited elevated C-reactive protein levels, while approximately one-third displayed high sedimentation rates and elevated platelet counts (38.5% and 39.8%, respectively). Additionally, low hemoglobin levels were noted in 41.9% of patients, with approximately two-thirds experiencing hypoalbuminemia (68.1%).

Patients with UC had a higher prevalence of low total protein levels compared to those with CD (25.6% vs. 3.9%; *p* = 0.034). Furthermore, when comparing CD and UC patients, reduced ferritin levels were more commonly observed in individuals with UC, whereas elevated ferritin levels were more prevalent in those with CD (*p* = 0.040) (Table 5).

Vitamin D deficiency was detected in 58.3% of patients, insufficiency in 31.7%, and only 10.0% of patients had optimal vitamin D serum concentrations. The median (IQR) vitamin D level was 38.2 nmol/L (27.2–59.9). It is noteworthy that children aged 12–18 had significantly lower median (IQR) vitamin D levels than those in the 6–12 age group (35.2 nmol/L (22.3–56.6) vs. 59.8 nmol/L (41.0–68.3), *p* = 0.012).

Folic acid deficiency was observed in 88.9% of patients, while none exhibited vitamin B12 deficiency. Clostridium difficile stool toxin tests yielded positive results in 14.8% of patients, whereas fecal calprotectin levels were elevated in nearly all patients (98.9%). The median (IQR) fecal calprotectin level exceeded 1554 µg/g (664.8–2243.5).

In addition to the previously mentioned higher frequency of extra-intestinal manifestations among children from rural areas, our analysis revealed no other significant differences among the afflicted individuals based on their urban or rural backgrounds. Similarly, there were no observed variations in the study participants based on gender.

Regarding treatment, corticosteroids were the primary therapy for 66 patients (61.7%) based on disease activity, while 18 UC patients initially received 5-aminosalicylic acid either orally or rectally. Maintenance treatment consisted of either azathioprine or 5-aminosalicylic acid, alongside nutritional support.

## 4. Discussion

In this retrospective study, we have presented medical records, including clinical data, diagnostic information, and both personal and family histories gathered during the first hospitalization of children diagnosed with IBD over a decade at the University Hospital of Split. Furthermore, we have reported a disease incidence rate as high as 9.9 per 100,000 children. When comparing patients with CD and UC, we have uncovered significant differences, including a later age at UC diagnosis and a shorter duration between the onset of initial symptoms and diagnosis. Additionally, while numerous studies suggest the influence of the environment in disease etiology, we did not find substantial variations concerning residential location, whether rural or urban, within this cohort. Despite the abundance of research on pediatric IBD, few studies comprehensively depicted extensive medical data from the moment of diagnosis.

Comparatively, the aforementioned incidence of IBD in the Split-Dalmatia County, Dalmatia, at 9.9 per 100,000 children, exceeded the 7.1 reported by Ivković et al. for the overall pediatric population in Croatia [31]. Additionally, the incidences of UC at 3.6 and CD at 4.2 were higher than those reported [31]. A systematic review has indicated that although data vary across regions, in some parts of Europe, pediatric IBD rates reach as high as 23 per 100,000 persons, while in North America, they exceed 15 per 100,000 persons [20,32]. Nevertheless, similar incidence rates to those in our study have been documented in neighboring Slovenia, as well as other European countries such as the Netherlands, Hungary, Norway, and France. While numerous studies continue to report an increase in incidence, some Western countries have reached a plateau in incidence rates [20,32].

Interestingly, in contrast to previous data pertaining to Croatia, our study revealed a higher incidence of CD compared to UC [31]. This discrepancy may potentially be attributed to the broader trend observed in the rising incidence of IBD in the northern hemisphere, primarily driven by an increase in CD cases, while the prevalence of UC remains relatively stable [18,20].

The predominance of affected male children compared to females was approximately 60%, with a male-to-female ratio of 1.5, slightly lower than the literature-reported 1.8 [17]. Additionally, there was no significant difference observed between UC and CD regarding the male-to-female ratios, which contradicted the findings of Kolaček et al.’s review, highlighting that while males are more prone to CD, there is no gender difference in UC [18]. However, as age advances, more females are affected, and these gender differences diminish, with the underlying reasons remaining unclarified [18].

In our investigation, the median age of children diagnosed with UC was 14.8 years, whereas those with CD were younger by more than 1 year, with a median age of 13.7 years. Consequently, a significantly higher percentage of children afflicted with UC (81%) were aged 12 or older compared to those with CD (68%). However, it is worth noting that literature data can be conflicting. In some countries, the increase in CD incidence is more pronounced among those under 10 years old, while elsewhere, it is more prominent in those over 10 years old [18]. Further, data suggest that UC is more common among Asians, Hispanics, and Turkish children compared to CD [33,34,35]. The reasons for the variations in IBD subtypes’ predominance, both overall and within specific age groups worldwide, remain to be elucidated [33,34]. The differentiation between UC and CD diagnoses was unfeasible for a single patient, accounting for 0.9% of cases, resulting in their classification as IBD-unclassified. In contrast, other studies have reported a higher prevalence of IBD-U [18,20,32,35]. This discrepancy could be attributed to our exclusive focus on patients during their initial hospitalization, allowing the possibility that, at the time of diagnosis, some patients clinically manifested features indicative of either CD or UC.

Consistent with previously mentioned findings, it comes as no surprise that we identified differences in disease incidence across age groups. In children aged 12 and older, the incidence was 19.7 per 100,000, while in those aged 6–12, it was nearly four times lower, at 5.8 per 100,000. A similar trend in Canada was observed by Benchimol et al. [36]. Only two of our patients were diagnosed under the age of six, preventing a detailed analysis and characterization of this specific group classified as very early onset IBD. Both patients had UC, and it is well-known that the disease course in this population can be more severe and refractory with children exhibiting a distinct phenotype. The aggressive nature of the disease and its early onset may suggest a significant genetic contribution to its development [26].

While a positive family history was observed in 8% of our patients, representing the most significant risk factor for disease development, numerous environmental factors also contribute to disease predisposition [37]. When comparing patients based on their residence, rural or urban, we did not identify significant differences among children, except for a higher frequency of extra-intestinal manifestations in those from rural regions. However, a direct comparison of incidence rates between urban and rural settings could not be conducted due to the unavailability of public data regarding the population under 18 years of age within specific territorial units. Nevertheless, given the established impact of certain environmental factors, primary prevention strategies, such as the rational use of antibiotics, promotion of breastfeeding, and avoidance of second-hand smoke exposure, are recommended for individuals with a positive family history [38].

The median duration of symptoms in our patients, which was 2 months, was relatively short, considering that most studies report an average diagnostic delay ranging from 2 to 4.5 months, with some even exceeding 8 months, as indicated by a systematic review [39]. The reasons for diagnostic delays are multifaceted, encompassing factors such as patients not disclosing symptoms to their parents, parents not promptly seeking medical attention for their children, and issues within the healthcare system itself, including specialist appointment delays and the time required for diagnosis by specialists [39]. Our study findings suggest that efforts have been made to minimize these factors, resulting in a shorter time to diagnosis. Furthermore, we demonstrated that the diagnostic delay was longer in CD patients compared to those with UC, possibly due to the more atypical and diverse clinical presentation associated with CD [20,33]. Regardless, our study identified several cases where the time to diagnosis exceeded one year, emphasizing the importance of raising public awareness about this disease and improving communication between primary pediatricians and hospital specialists [39].

In our study, the most prevalent symptoms among children with IBD were classic GI disturbances, including diarrhea, abdominal pain, and rectal bleeding [17]. Numerous other GI symptoms were also observed, although less frequently, indicating that the disease’s presentation can be nonspecific. Thus, it is essential to consider IBD when managing pediatric patients with digestive tract conditions [17]. In line with the existing literature, there was a statistically significant over twofold higher occurrence of rectal bleeding in UC patients compared to those with CD. Moreover, despite weight loss being more typical in children with CD, no significant difference was observed among our subjects [17,35].

Additionally, less than 2% of the participants exhibited severe short stature, while approximately 12% were either underweight or severely underweight. As reported by Selbuz et al., the rate of growth impairment increases over time from the diagnosis, especially during the active stages of the disease, where the nutritional status deteriorates [40]. Nutritional deficits in IBD are primarily attributed to factors such as anorexia, insufficient caloric intake, malabsorption, and chronic inflammation [41]. This underscores the vital role of nutritionists as integral members of the medical team responsible for treating and caring for these patients. Their involvement provides opportunities for implementing various therapeutic approaches that prioritize nutrition, either as a primary treatment or to address nutritional deficiencies and malnutrition [41]. Apart from malnutrition, less than 7% of children were overweight, which contrasts with over one-fifth of American pediatric IBD patients [35,42]. Nevertheless, it is important to emphasize that obesity was previously associated with disease activity, higher complication rates, increased morbidity, and mortality in IBD patients [42].

Among the associated diseases identified in a quarter of our study participants, celiac disease (CeD) was present in 5.6% of them. This aligns with the existing literature suggesting a tenfold higher incidence of IBD development in individuals with CeD compared to the general population [10]. Recent research has highlighted a bidirectional causal relationship between CeD and IBD, particularly CD, while UC demonstrates a unidirectional association, increasing the risk of CeD development. This intriguing interrelationship suggests the potential sharing of common genetic, immunological, and environmental risk factors that contribute to altered immune responses and disease development [10]. These findings emphasize the importance of clinical vigilance in diagnosing co-occurring CeD and IBD, as patients with both conditions are more likely to experience IBD-related complications. Moreover, this link between the two conditions could shed light on previously unclear pathophysiological mechanisms and underscores the necessity for developing novel therapeutic approaches for patients with comorbid diseases. This, in turn, holds promise for improved disease prevention through ongoing research [10].

Laboratory biomarkers, despite their general lack of specificity, offer valuable insights into disease progression and treatment response, warranting their routine inclusion in clinical assessments [11]. C-reactive protein (CRP), renowned for its remarkable sensitivity in detecting inflammation, exhibited elevated levels in 71% of our study participants. Nonetheless, the non-specific nature of CRP presents a limitation, as it can increase in reaction to diverse tissue damages. Consistent with our findings, CRP produced negative results in 30% of patients with endoscopically active CD and demonstrated an inability to reliably distinguish between UC and CD [11].

Additionally, fecal calprotectin, which was elevated in nearly all participants with a median of approximately 1550 µg/g, serves as a direct biomarker of intestinal inflammation [10]. While boasting relatively high sensitivity and specificity, these metrics vary across studies depending on the chosen cutoff values [12]. Values exceeding 250 µg/g should certainly raise suspicion of underlying inflammation, and this parameter can aid in distinguishing between IBD and irritable bowel syndrome [11,12]. Although fecal calprotectin may not suffice as a predictor of endoscopic and histologic remission in disease monitoring, it remains a valuable tool, particularly in cases where endoscopic assessments are unfeasible [12].

We identified a prevalence of vitamin D deficiency in 58.3% of the patients, which exceeded the 38% reported by Syed et al. but aligned closely with the 60% figure found by Śledzińska et al. [43,44]. When considering both vitamin D insufficiency and deficiency, only 10% of patients achieved the target vitamin D concentration, in line with findings from previous investigations [43,44,45]. A study by Karin et al. in Croatia reported a 58% prevalence of vitamin D deficiency among healthy preschool children, a figure that closely mirrored the 58.3% rate observed in children diagnosed with IBD in our study [46]. Vitamin D has been a focal point in various studies involving children with IBD and its multifaceted role in immune-mediated diseases, including its immunomodulatory effects on pro-inflammatory cytokines, epithelial barriers, and systemic immune cells, has been well-established. However, its specific impact on the course of IBD remains unconfirmed. Despite this, the majority of clinicians recommend routine monitoring of vitamin D levels and supplementation in cases of deficiency [47].

The limitations of this study should be considered. First, due to its retrospective nature and the study’s structure, certain laboratory parameters and anthropometric measurements were not conducted for all patients, resulting in incomplete data for these characteristics. The specific instances where data were missing have been highlighted. Second, since only the first hospitalization was analyzed, follow-up data on the disease’s progression, outcomes, and potential changes in diagnosis within the spectrum of IBD were not included. Third, given that the study was conducted at a single center, minor differences among the patients may not have been fully captured, highlighting the need for further research involving a larger sample size. To mitigate this impact, we analyzed a ten-year period, encompassing a more significant number of affected individuals.

## 5. Conclusions

The incidence of IBD in children in Split-Dalmatia County exceeds that in the general Croatian pediatric population, with a male predominance. While there are distinctions in the pathophysiology between UC and CD, our study highlights their shared characteristics, underscoring the limited utility of laboratory, anamnestic, and clinical data for differentiation. Endoscopic and histopathological evaluation remains the primary diagnostic approach. Significant differences between CD and UC patients include older age in UC, shorter diagnostic delays, higher rates of rectal bleeding in UC, absence of perianal manifestations, and reduced prevalence of elevated serum ferritin levels. Despite the influence of environmental factors on IBD incidence, our findings indicate that, except for a higher frequency of extra-intestinal manifestations, there are no disparities between children with IBD based on their rural or urban residence.

## Figures and Tables

**Table 1:** Demographic characteristics of patients according to type of inflammatory bowel disease (IBD); median (interquartile range) or *n* (%).

Parameters	Total IBD (*n* = 107)	UC (*n* = 47)	CD (*n* = 59)	*p*-Value
Age (years)	14.1 (11.6–16.1)	14.8 (12.8–16.7)	13.7 (11.4–15.8)	0.044 *
<6	2 (1.9)	2 (4.3)	0 (0.0)	0.043 ^†^
6–<12	26 (24.3)	7 (14.9)	19 (32.2)	
12–<18	79 (73.0)	38 (80.9)	40 (67.8)	
Symptoms duration (months)	2.0 (1.0–5.0); *n* = 97	2.0 (0.0–3.0); *n* = 42	3.0 (2.0–6.0); *n* = 55	0.003 *
Gender				
Male	65 (60.7)	28 (59.6)	37 (62.7)	0.742 ^†^
Female	42 (39.3)	19 (40.4)	22 (37.3)	
Residence				
Urban	77 (72.0)	33 (70.2)	43 (72.9)	0.762 ^†^
Rural	30 (28.0)	14 (29.8)	16 (27.1)	
Family history among first- and/or second-degree relatives				
Autoimmune disorders	23 (21.7)	11 (23.4)	12 (20.3)	0.704 ^†^
IBD	9 (8.4)	4 (8.5)	5 (8.5)	0.995 ^†^
Other autoimmune disorders	19 (17.9)	10 (21.3)	9 (15.3)	0.422 ^†^

* Mann–Whitney U test, ^†^ Chi-squared test, UC: ulcerative colitis, CD: Crohn’s disease.

**Table 2:** Inflammatory bowel disease (IBD) incidence rates per 100,000 children/year in Split Dalmatia County, Croatia.

Patient Group	Incidence Rate; Median (95% Confidence Interval)	*p*-Value
Total IBD ^‡^	9.89 (5.93–13.84)	
Type of IBD ^‡^		
Ulcerative colitis	3.63 (2.29–6.42)	0.345 *
Crohn’s disease	4.21 (2.53–8.29)	
Age (years) ^§^		
<6	0.00 (0.00–1.96)	<0.001 ^†^
6–<12	5.75 (3.52–10.73)	
12–<18	19.68 (11.87–26.42)	
Gender ^§^		
Male	11.19 (5.74–14.98)	0.174 *
Female	8.67 (4.69–10.94)	

* Mann–Whitney U test, ^†^ Kruskal–Wallis test, ^‡^ estimated for total children population, ^§^ estimated for specific demographic groups.

**Table 3:** Anthropometric characteristics of children according to type of inflammatory bowel disease (IBD); median (interquartile range) or *n* (%).

Parameters	Total IBD (*n* = 105)	UC (*n* = 45)	CD (*n* = 59)	*p*-Value
Height (z-score)	0.70 (0.04–1.29)	0.75 (0.17–1.37)	0.70 (0.04–1.29)	0.903 *
Severe short stature	2 (1.9)	2 (4.4)	0 (0.0)	-
Weight (z-score)	0.22 (−0.56–0.76)	0.25 (−0.51–0.88)	0.22 (−0.62–0.72)	0.533 *
BMI (z-score)	−0.34 (−0.97–0.45)	−0.21 (−1.10–0.57)	−0.45 (−0.95–0.40)	0.383 *
Normal weight	85 (81.0)	35 (77.8)	49 (83.1)	0.874 ^†^
Overweight	7 (6.7)	4 (8.9)	3 (5.1)	
Underweight	11 (10.5)	5 (11.1)	6 (10.2)	
Severe underweight	2 (1.9)	1 (2.2)	1 (1.7)	

* Mann–Whitney U test, ^†^ Chi-squared test, UC: ulcerative colitis, CD: Crohn’s disease, BMI: body mass index.

**Table 4:** Clinical presentation of patients according to type of inflammatory bowel disease (IBD); *n* (%).

Parameters	Total IBD (*n* = 107)	UC (*n* = 47)	CD (*n* = 59)	*p*-Value
Diarrhea	65 (60.7)	33 (70.2)	32 (54.2)	0.093 *
Abdominal pain	54 (50.5)	20 (42.6)	33 (55.9)	0.171 *
Rectal bleeding	53 (49.5)	34 (72.3)	19 (32.2)	<0.001 *
Fever	17 (15.9)	5 (10.6)	12 (20.3)	0.176 *
Weight loss	15 (14.0)	4 (8.5)	11 (18.6)	0.137 *
Nausea	8 (7.5)	2 (4.3)	6 (10.2)	0.252 *
Growth retardation	3 (2.8)	2 (4.3)	1 (1.7)	0.430 *
Moth ulcers	3 (2.8)	0 (0.0)	3 (5.1)	0.177 *
Tenesmus	3 (2.8)	2 (4.3)	1 (1.7)	0.430 *
Appendicitis	2 (1.9)	0 (0.0)	2 (3.4)	-
Constipation	2 (1.9)	1 (2.1)	1 (1.7)	-
Lethargy	2 (1.9)	0 (0.0)	2 (3.4)	-
Inappetence	1 (0.9)	0 (0.0)	1 (1.7)	-
Incontinence	1 (0.9)	0 (0.0)	1 (1.7)	-
Heartburn	1 (0.9)	0 (0.0)	1 (1.7)	-
Meteorism	1 (0.9)	0 (0.0)	1 (1.7)	-
Extra-intestinal manifestations	19 (17.8)	7 (14.9)	11 (18.6)	0.609 *
Dermatologic	2 (1.9)	1 (2.1)	1 (1.7)	-
Musculoskeletal	9 (8.4)	4 (8.5)	5 (8.5)	0.995 *
Hepatic	3 (2.8)	1 (2.1)	1 (1.7)	-
Hematologic	1 (0.9)	0 (0.0)	1 (1.7)	-
Aphtous stomatitis	3 (2.8)	1 (2.1)	2 (3.4)	0.697 *
Pericardial effusion	1 (0.9)	0 (0.0)	1 (1.7)	-
Other	1 (0.9)	0 (0.0)	1 (1.7)	-
Associated diseases	28 (26.1)	14 (29.8)	14 (23.7)	0.482 *
Celiac disease	6 (5.6)	2 (4.3)	4 (6.8)	0.576 *
Respiratory diseases	5 (4.7)	4 (8.5)	1 (1.7)	0.100 *
Chronic gastritis	2 (1.9)	1 (2.1)	1 (1.7)	-
Valvular dysfunction	5 (4.7)	3 (6.4)	2 (3.4)	0.470 *
Other	12 (11.2)	4 (8.5) ^†^	8 (13.6) ^‡^	0.415 *
Allergies	12 (11.2)	6 (12.8)	6 (10.2)	0.675 *
Perianal manifestations	8 (7.5)	0 (0.0)	8 (13.6)	0.009 *

* Chi-squared test, ^†^ Pervasive developmental disorder, hemangioma of the knee, Klinefelter sy, emotional disturbance, ^‡^ Dermatitis, varicocele, urinary incontinence, pilonidal sinus disease, fatty acid beta-oxidation disorder, developmental delay, scoliosis, liver lesion, UC: ulcerative colitis, CD: Crohn’s disease.

**Table 5:** Laboratory abnormalities according to type of inflammatory bowel disease (IBD); *n*/*n* (%).

Parameters	Total IBD	UC	CD	*p*-Value
Elevated CRP	72/101 (71.3)	33/44 (75.0)	39/56 (69.6)	0.554 *
High sedimentation rate	30/78 (38.5)	11/33 (33.3)	19/44 (43.2)	0.380 *
Leukocytosis	35/104 (33.7)	19/45 (42.2)	16/58 (27.6)	0.120 *
Low hemoglobin	44/105 (41.9)	22/46 (47.8)	22/58 (37.9)	0.310 *
Elevated platelets	41/103 (39.8)	18/45 (40.0)	23/57 (40.4)	0.971 *
Hypoalbuminemia	64/94 (68.1)	27/39 (69.2)	37/54 (68.5)	0.942 *
Low total protein	15/94 (16.0)	10/39 (25.6)	5/54 (3.9)	0.034 *
Low iron	39/89 (43.8)	16/35 (45.7)	23/53 (43.4)	0.830 *
Low ferritin	27/86 (31.4)	13/34 (38.2)	13/51 (25.5)	0.040 *
Elevated ferritin	16/86 (18.6)	2/34 (5.9)	14/51 (27.5)	
Vitamin D deficiency	35/60 (58.3)	22/33 (66.7)	13/27 (48.1)	0.331 *
Vitamin D insufficiency	19/60 (31.7)	8/33 (24.2)	11/27 (40.7)	
Low folic acid	24/27 (88.9)	7/9 (77.8)	17/18 (94.4)	0.194 *
Low vitamin B12	0/36 (0.0)	0/13 (0.0)	0/23 (0.0)	-
Elevated IgG levels	18/31 (58.1)	4/9 (44.4)	14/22 (63.6)	0.326 *
Elevated IgE levels	4/23 (17.4)	3/13 (23.1)	1/10 (10.0)	0.412 *
Elevated TSH	2/15 (13.3)	0/9 (0.0)	2/6 (33.3)	-
Positive Clostridium difficile stool toxin test	8/54 (14.8)	5/31 (16.1)	3/23 (13.09	0.752 *
Elevated fecal calprotectin	89/90 (98.9)	44/44 (100.0)	44/45 (97.8)	-

* Chi-squared test, UC: ulcerative colitis, CD: Chron’s disease, CRP: C-reactive protein, TSH: thyroid stimulating hormone.

## Data Availability

The data presented in this study are available on request from the corresponding author.
